# Supplementary material for: Blind demixing methods for recovering dense neuronal morphology from barcode imaging data
Source: PLoS Comput Biol. 2022 Apr 8;18(4):e1009991. doi: 10.1371/journal.pcbi.1009991 (PMC9020678; doi:10.1371/journal.pcbi.1009991)
Supplement: S1 Appendix — (PDF) [file pcbi.1009991.s001.pdf]

Blind demixing methods for recovering dense neuronal morphology from barcode imaging data
S1 Appendix

Shuonan Chen[1,2,3,4,5,6*], Jackson Loper[1,2,3,4,5,7], Pengcheng Zhou[8,9], and Liam Paninski[1,2,3,4,5,7]

[1]Mortimer B. Zuckerman Mind Brain Behavior Institute, Columbia University, New York, New York, United States of America
[2]Department of Statistics, Columbia University, New York, New York, United States of America
[3]Center for Theoretical Neuroscience, Columbia University, New York, New York, United States of America
[4]Grossman Center for the Statistics of Mind, Columbia University, New York, New York, United States of America
[5]Department of Neuroscience, Columbia University, New York, New York, United States of America
[6]Department of Systems Biology, Columbia University, New York, New York, United States of America
[7]Data Science Institute, Columbia University, New York, New York, United States of America
[8]Faculty of Life and Health Sciences, Shenzhen Institute of Advanced Technology, Chinese Academy of Sciences, Shenzhen, China
[9]The Brain Cognition and Brain Disease Institute, Shenzhen Institute of Advanced Technology, Chinese Academy of Sciences, Shenzhen, China
[*]sc4417@cumc.columbia.edu

A Detailed methods

A.1 Data simulation

The simulation process is illustrated in Fig 2. Specifically, we started with all the cells from a selected region of brain, densely-reconstructed from the MICrONS electron microscopy dataset (cf. MICrONS Explorer [1–3]). A $(4 \times 4 \times 4)$ μm region is shown in Fig 2. These shapes were originally stored as triangular meshes; we converted them to binary 2D or 3D voxel tensors, using the voxels with pre-specified size under each simulation setting (the original MICrONS data was 3.58×3.58 nanometer resolution with 40 nanometer thick sections; in Fig 2 we show $(100 \times 100 \times 100)$-nanometer voxel size, as in the high-resolution simulations).

For each neuron we also created a unique 'one-hot' barcode, assuming R rounds of imaging with C channels. That is, for each neuron j we constructed a random binary matrix $\mathbf{B}_j \in \mathbb{R}^{R \times C}$ such that each row contains exactly one nonzero entry. Assuming $C = 4$ color channels, this enables labeling of 4^R unique neurons; we used $R = 17$ in all our simulations. Three examples of simulated barcodes can be seen in the top right panel in Fig 2.

Our assumption that each cell carries a unique barcode merits discussion, on two points. First, two neurons might be labeled by the same barcode, or very similar barcodes. This potential issue was discussed in a recent review [4], where it is shown that the fraction of uniquely labeled cells approximates to 1 when the number of barcodes is sufficiently large relative to the number of cells that are labeled. Since in our simulation study, the number of possible barcodes (4^{17}) is much larger than the number of cells, we concluded that it was safe to assume that every neuron under study here is uniquely labeled. Indeed, after the barcodes are uniformly sampled, we found that the minimum Hamming distance between any pair of barcodes is at least 8, which is enough to easily distinguish one from the others in the imagestack. Second, a single neuron might be labeled by two distinct barcodes ('double-infection'), though previous work indicates this is rare [5]. For simplicity we here ignored this possibility; if double-infection were a significant problem, it would be necessary to implement an algorithm to match pseudocolors together if they trace out similar paths. We leave this for future work.

To construct the imagestack itself, we input the transcript locations and codebook into Algorithm 1. This algorithm attempts to replicate the physical processes by which transcripts in tissue become fluorescent signals which are captured by a camera. It includes parameters to control many features of this process, including the variability in overall signal strength of each transcript, variability in per-round signal strength, a point spread function (PSF), and random per-voxel 'speckle noise.' We set 'signal_range' to be (10, 15) (randomly scaling the overall signal strength of each amplicon between 10 and 15). We set 'per_frame_signal_range' to be (0.8, 1) (randomly rescaling the signal in each frame independently for each amplicon between 0.8 and 1.0). We based these parameters on manual investigation of recent experimental data [6]. We used several different choices for 'blursz,' depending on the dataset simulation details (see the following subsections).

A.1.1 High-resolution, dense-expression simulation

For the high-resolution simulation setting, for every neuron in a selected region of the brain (one $(14.6 \times 14.6 \times 19.7)$ μm field of view (FOV) from the MICrONS dataset), we first voxelized the mesh objects using $(100, 100, 100)$ nm voxels. Then we simulated the locations of amplicons (barcoded RNA transcripts) inside that voxelized neuron. These locations were sampled from a homogeneous Poisson process with rate λ. This rate constant has units of 'density per cubic microns'; it indicates the average number of amplicons one would expect to find in a given cubic micron of neuronal tissue. See Fig 3 for examples of several imagestacks simulated with different densities λ. If λ is too low, the barcode signal will be too sparse to reconstruct or even detect cells; if λ is too high, the signal could become overwhelmingly mixed during imaging, making it difficult to identify the boundaries between multiple neurons in close proximity. See Fig 4 for an illustration of this density issue, even with a medium amplicon density.

Once we have determined the numbers and the locations of these amplicons, we applied a PSF to the images to mimic the image obtained from light microscopy; we used a Gaussian kernel with standard deviation of size $(200, 200, 200)$ nm, blurring the signal by roughly 2 voxels in all directions.

Algorithm 1: create_imagestack

input : locations, codebook, speckle_noise, signal_range, per_frame_signal_range, blursz
output: $\mathbf{X} \in \mathbb{R}^{R\times C\times M_0\times M_1\times M_2}$ – a simulated stack of images representing R rounds of spatial transcriptomics images using C colors over a $(M_0 \times M_1 \times M_2)$-voxel region

$\mathbf{X} \leftarrow 0$; `// initialize imagestack`
foreach *transcript located at position* m_0, m_1, m_2 *with barcode* $\mathbf{B}_j$ **do**
  $\tilde{\mathbf{B}}_j \leftarrow$distort_barcode($\mathbf{B}_j$, signal_range, per_frame_signal_range);
  foreach r,c **do**
    $\mathbf{X}_{r,c,m_0,m_1,m_2} \leftarrow \mathbf{X}_{r,c,m_0,m_1,m_2} + \tilde{\mathbf{B}}_{j,r,c}$;
  end
end
foreach r,c **do**
  $\mathbf{X}_{r,c} \leftarrow$gaussian_blur($\mathbf{X}_{r,c}$,blursz) ; `// apply PSF`
end
$\mathbf{X} \sim \mathcal{N}(\mathbf{X}$, speckle_noise) ; `// add speckle noise`
$\mathbf{X} \leftarrow (0 \vee \mathbf{X})$; `// insist on nonnnegative values`

Algorithm 2: distort_barcode

input : $\boldsymbol{\beta} \in \mathbb{R}^{R\times C}$, signal_range, per_frame_signal_range
output: $\tilde{\boldsymbol{\beta}} \in \mathbb{R}^{R\times C}$ – a distorted barcode

signal_mult $\leftarrow$ random(signal_range);
foreach r,c **do**
  $\tilde{\boldsymbol{\beta}}_{r,c} \leftarrow \boldsymbol{\beta}_{r,c} \times$ signal_mult ; `// overall strength scaling for this transcript`
  $\tilde{\boldsymbol{\beta}}_{r,c} \leftarrow \tilde{\boldsymbol{\beta}}_{r,c} \times$ random(per_frame_signal_range) ; `// scaling for each frame`
end

	Description	Dimensions	Support
M_0, M_1, M_2	number of voxels in each dimension (product is M)	scalar	$\mathbb{N}$
C	number of types of probes/wavelengths	scalar	$\mathbb{N}$
R	number of imaging rounds	scalar	$\mathbb{N}$
J	number of barcodes used to label neurons	scalar	$\mathbb{N}$
$\mathbf{X}$	simulated imagestack	$R \times C \times M$	$\mathbb{R}^+$
$\mathbf{B}$	binary codebook matrix	$J \times R \times C$	$\{0,1\}$
$\tilde{\mathbf{B}}$	distorted binary codebook matrix	$J \times R \times C$	$\{0,1\}$
$\boldsymbol{\beta}$	binary codebook for a particular cell	$R \times C$	$\{0,1\}$
$\tilde{\boldsymbol{\beta}}$	distorted binary codebook for a particular cell	$R \times C$	$\{0,1\}$
`speckle_noise`	noise added to the simulated imagestack	scalar	$\mathbb{R}$
`signal_range`	range of the intensity for each frame, each amplicon	vector	$\mathbb{R}^+$
`per_frame_singal_range`	fluctuations on the signal strength for each frame	vector	$[0,1]$
`blursz`	size of PSF in units of voxels	vector	$\mathbb{N}$
`round_satisfaction_threshold`	threshold for a channel to be considered to be active	scalar	$[1,\infty)$

Table 1: Notation

A.1.2 Low-resolution, sparse-expression simulation

For the low-resolution simulation, the simulation process was similar to the high-resolution case described above, with some modifications. The first difference is that instead of studying one FOV, we started from a larger region of the brain (of size $(196, 129, 40)$ μm, which is the full region used in [3]). Second, the voxel size used to convert the original mesh data was $(5 \times 5 \times 40)$ μm, and the PSF was not added for this simulation

since the optical resolution is so low in this setting. The number of amplicons in a neuron is considered to be proportional to its axon length (amplicons per micron length), instead of volume of the axons (amplicons per cubic micron): we used 0.08 amplicons per μm as the density of the simulated amplicons, matching [7]. Also, only the axonal segments are considered, and we used a simple criterion to remove dendrites (described in A.3.5). This is because in this setting we are simulating the tracing of long-range neuronal projections, and therefore restricted our focus to only the axonal parts of the neuronal segment rather than dendritic parts, even though the original EM data included all the neurons within the selected region. Lastly, only a small proportion of neurons were modeled to be infected by the virus (i.e., labeled by the barcodes). We randomly selected only 1% of all the neurons from the EM images (a more realistic assumption based on current cellular barcoding technologies [7]). An example of the resulting simulated data is shown in the left panel of Fig 10.

A.2 Barcode estimation

Identifying the barcodes present in a given region of tissue is straightforward in settings with high optical resolution and low labeling density, because we can take advantage of the one-hot nature of the barcodes: each barcode is designed so that in each round exactly one channel is illuminated. On the other hand, for low-resolution and/or densely-labeled datasets, a more sophisticated iterative approach is necessary. We outline the main ideas below.

A.2.1 Naïve barcode library estimation

If a voxel contains signal dominated by only one barcode, exactly one channel should be brightest in each imaging round. This suggests the following procedure: scan for voxels that are 'nearly one-hot' — i.e., in each round there is a single channel which is much brighter than all others — and use the bright channel in each round to estimate the barcode for this voxel.

To make this idea practical, there are several difficulties that must be overcome. First, we need to find a way to look at the fluorescence data and decide when a voxel is 'nearly one-hot.' For this it is necessary to pick a threshold value. Second, we need a way to integrate information across the entire tissue. We can collect barcode candidates from a modestly large region of tissue, but each barcode candidate may be corrupted by noise from the data, yielding many almost-identical barcodes that are all slightly incorrect. To get the best possible barcode estimates, we need a way to merge similar barcodes together.

We adopt the following strategy. At each voxel location m and for each round r we identified the brightest channel, $c^*_{r,m} = \arg\max_c \mathbf{X}_{r,c,m}$. We also identify whether this brightest channel is significantly above the other channels. Using this information we produce an initial estimate of a barcode that might be present in this voxel. These initial barcodes are defined by thresholding:

$$\tilde{\mathbf{B}}_{m,r,c} = \begin{cases} 1 & \text{if } c = c^*_{r,m} \text{ and } \mathbf{X}_{r,c^*_{r,m},m} \geq \text{round\_satisfaction\_threshold} \times \frac{1}{R}\sum_r \mathbf{X}_{r,c,m} \\ 0 & \text{if } c \neq c^*_{r,m} \text{ and } \mathbf{X}_{r,c^*_{r,m},m} \geq \text{round\_satisfaction\_threshold} \times \frac{1}{R}\sum_r \mathbf{X}_{r,c,m} \\ \text{nan} & \text{otherwise} \end{cases}$$

Here, a 'nan' indicates that we are uncertain about the barcode signal for this location at this round; i.e., the signal from the brightest channel ($c^*_{r,m}$) is not high enough for us to confidently determine this is the correct channel. 'Round_satisfaction_threshold' is set to be 1 (i.e., the bright channel must be higher than the average of all the rounds) in the results presented here. We compared the signal strength in these bright channels with the total signal strength, according to the formula

$$\text{ratio}_m = \frac{\sum_{rc:\ \tilde{\mathbf{B}}_{m,r,c}=1} \mathbf{X}^2_{r,c,m}}{\text{signal\_control} + \sum_{rc} \mathbf{X}^2_{r,c,m}}.$$

Here 'signal_control' is a parameter that controls our sensitivity to voxels with low overall strength; unless total signal strength in $\mathbf{X}$ at voxel m is significant, this term will cause the overall ratio to become small. For each voxel location m, if ratio_m is higher than a threshold, we conclude there is a valid amplicon at this voxel location.

We construct an initial codebook by concatenating the codebooks associated with all the voxels where the ratio discussed above exceeds a threshold. We then attempt to remove duplicates from this codebook. We first merge any barcodes that are exactly identical. We then use a greedy algorithm to merge any barcodes which are within a given Hamming distance (we chose a minimal distance of 3 here, as we found that amplicon locations could still be accurately estimated with this level of corruption). Barcodes are merged according to the formula

$$\text{merge}(\tilde{\mathbf{B}}_m, \tilde{\mathbf{B}}_{m'})_{r,c} = \begin{cases} \tilde{\mathbf{B}}_{m,r,c} & \text{if } \tilde{\mathbf{B}}_{m',r,c} = \text{nan} \\ \tilde{\mathbf{B}}_{m',r,c} & \text{if } \tilde{\mathbf{B}}_{m,r,c} = \text{nan} \\ \tilde{\mathbf{B}}_{m,r,c} & \text{otherwise.} \end{cases}$$

Note that this approach uses the first barcode's information if there is disagreement: the order in which barcodes are merged could make a difference. So far this has not introduced any significant problems, but there are alternatives that could be pursued in the future (e.g., ordering so that the brightest barcodes come first).

Henceforth we will use $\mathbf{B}$ to denote the final codebook yielded by the approach above, with $\mathbf{B}_{j,r,c}$ denoting the value for barcode j in round r and channel c. We will let J denote the total number of barcodes identified (after deduplication).

A.2.2 Iterative barcode discovery and amplicon location estimation

In low-resolution or densely-labeled settings, the naïve approach outlined above was not sufficient. There were many barcodes which did not appear alone in any voxel, making it essentially impossible to identify them using the method above. In this case, we found the following iterative procedure effective.

We first identify some barcodes in the imagestack, using the naïve approach given above. We then estimate signal arising from those barcodes, and attempt to remove it from the imagestack. We then apply the naïve approach again to the residual imagestack to find additional barcodes. Next, we return to the original imagestack, and attempt once again to estimate all signal arising from any barcode that we have discovered. This procedure, formalized in Algorithm 3, can be repeated until no new barcodes are discovered (or for a prespecified number of iterations).

Algorithm 3: iterative_barcode_and_amplicon_estimation

input : $\mathbf{X}$, $\mathbf{N}$, signal_control, round_satisfaction_threshold
output: $\mathbf{B} \in \mathbb{R}^{J \times R \times C}$ – the final barcodes found

$\mathbf{X}' \leftarrow \mathbf{X}$;
$\mathbf{B} \leftarrow$ *empty*;
for $i \leftarrow 1$ *to* $\mathbf{N}$ **do**
  $\tilde{\mathbf{B}}^{(i)} \leftarrow$ barcode_discover($\mathbf{X}'$, signal_control, round_satisfaction_threshold) ; `// described in Section A.2.1`
  $\tilde{\mathbf{B}}^{(i)} \leftarrow$ barcode_deduplication($\tilde{\mathbf{B}}^{(i)}$) ; `// described in Section A.2.1`
  if $\tilde{\mathbf{B}}^{(i)}$ is not empty **then**
    $\mathbf{B} \leftarrow$ merge($\mathbf{B}, \tilde{\mathbf{B}}^{(i)}$) ; `// described in Section A.2.1`
    recon $\leftarrow$ BarDensr_underapprox_reconstruction($\mathbf{X}, \mathbf{B}$);
    $\mathbf{X}' \leftarrow \max\Big(0, \mathbf{X} - \text{recon}\Big)$; `// get the non-negative residual`
  end
end

To enact this algorithm, we need a way to estimate all signal in an imagestack arising from a particular set of barcodes, *without* full knowledge of the true barcode library. For this task we build on the BarDensr model [8], which demixes and deconvolves spatial transcriptomic imagestacks by modeling the physical process that gives rise to the observed fluorescences.

We assume the following non-negative matrix factorization model for the observed data, $\mathbf{X}$:

$$\mathbf{X}_{r,c,m} \approx \sum_j \mathbf{B}'_{r,c,j} \mathbf{F}'_{m,j} + \sum_j \hat{\mathbf{B}}_{r,c,j} \hat{\mathbf{F}}_{m,j}.$$

Here $\hat{\mathbf{B}}$ represents the set of barcodes which have already been discovered, $\hat{\mathbf{F}}$ indicates a non-negative fluorescent intensity for each known barcode at each voxel, $\mathbf{B}'$ represents the set of barcodes which are yet undiscovered, and $\mathbf{F}'$ indicates a non-negative fluorescent intensity for each unknown barcode at each voxel. Note that we do not expect the equation above to hold exactly, due to various forms of noise, but we hope that it will be approximately correct. This equation can be seen as a version of BarDensr model, generalized to the case that some barcodes are unknown.

Under this model, the signal from the known barcodes corresponds to $\sum_j \hat{\mathbf{B}}_{r,c,j}\hat{\mathbf{F}}_{m,j}$. To estimate this signal it is thus sufficient to estimate $\hat{\mathbf{F}}$. This problem is difficult because of the the unknown barcodes contributing signal to the observed imagestack $\mathbf{X}$. Fortunately, we can make use of the non-negativity constraints of the model to help. From these constraints, it follows that

$$\sum_j \hat{\mathbf{B}}_{r,c,j}\hat{\mathbf{F}}_{m,j} \approx \mathbf{X}_{r,c,m} - \sum_j \mathbf{B}'_{r,c,j}\mathbf{F}'_{m,j} \leq \mathbf{X}_{r,c,m}.$$

We call this the 'under-approximation' constraint, because it ensures that the estimated signal arising from the known barcodes is less than the observed fluoresence.

Now we need to choose an appropriate loss function. One approach would be to find $\hat{\mathbf{F}}$ by minimizing the mean squared error between $\mathbf{X}_{r,c,m}$ and $\sum_j \hat{\mathbf{B}}_{r,c,j}\hat{\mathbf{F}}_{m,j}$; this would be similar to the approach taken in [8], where a quadratic objective together with a simple non-negative constraint could be efficiently optimized using standard techniques from the non-negative matrix factorization literature. However, adding the under-approximation constraint leads to more generic kinds of quadratic programming problems which are expensive to solve when the number of voxels is large. By slightly modifying this objective, we can obtain a fairly easy linear programming problem: instead of minimizing the mean squared error, we maximize the dot-product between the data and the estimated signal from the known barcodes. Thus we want to account for as much of the observed fluorescence as possible without overstepping the constraints. (Mathematically, we have replaced a quadratic penalty on the reconstruction with a linear penalty and a set of linear constraints that bound the magnitude of the reconstruction at each voxel.)

Putting the constraints together with the loss function, we arrive at the following approach for estimating the signal from the known barcodes. We first solve the following linear programming problem.

$$\begin{aligned} \max_{\hat{\mathbf{F}}} \quad & \sum_{r,c,m,j}^{R,C,M,J} \mathbf{X}_{r,c,m}\hat{\mathbf{B}}_{r,c,j}\hat{\mathbf{F}}_{m,j} \\ \text{subject to} \quad & \hat{\mathbf{F}}_{m,j} \geq 0 \quad \forall m, j \\ & \mathbf{X}_{r,c,m} \geq \sum_j^J \hat{\mathbf{B}}_{r,c,j}\hat{\mathbf{F}}_{m,j} \quad \forall r, c, m \end{aligned}$$

We then take $\sum_j \hat{\mathbf{B}}_{r,c,j}\hat{\mathbf{F}}_{m,j}$ as our estimate of the signal arising from the known barcodes. We call this problem the 'BarDensr_underapprox' problem. This new problem enforces non-negativity in two ways: it enforces that $\hat{\mathbf{F}}_{m,j}$ is non-negative but it also enforces that $\mathbf{X}_{r,c,m} - \sum_j \hat{\mathbf{B}}_{r,c,j}\hat{\mathbf{F}}_{m,j}$ is non-negative. This approach is fairly fast, especially with parallel hardware such as GPUs, because the main computational effort comprises linear programming problems which can all be solved independently. We found we could make it faster still by assuming that only a small subset of the fluorescence densities were nonzero at each voxel. In particular, at each voxel we computed the correlation between that voxel and each barcode; we then assumed that only nine barcodes with the largest correlations carried nonzero fluorescent densities. This approximation yielded essentially no difference in the final results on any data that we tried it on. In practice, we found we could solve the 'BarDensr_underapprox' problem about six times faster than the original quadratic BarDensr optimization problem (16 seconds versus 96 seconds for a (30,30,30)-voxel patch using an Nvidia T4 GPU).

We found this constrained optimization approach to be effective even when our estimate of the barcode library represents a small fraction of the true set of barcodes which give rise to the imagestack. We also investigated a variant of this method which does not use the under-approximation constraint; this variant was unsuccessful. This finding adds to a growing body of work in the non-negative matrix factorization literature that shows that imposing under-approximation leads to robustness in the face of unknown contaminating signals [9, 10].

A.2.3 Iterative barcode discovery for the real data

The 'BarDensr_underapprox' problem described in the last section assumes that the amplicon signal is even across frames. However, because of the large frame-wise variation of the signal intensity on the real experimental data, directly applying the above method would result in overly underestimated reconstruction. Therefore, we estimated the frame-wise signal intensity based on the detected spots and incorporated this information while reconstructing the original images at each iteration. Specifically, this was done by running BarDensr after finding the barcodes in each iteration, to estimate the per-round per-channel scale factor ($\alpha \in \mathbb{R}^{R\times C}$ in the original publication), which quantifies the amplicon signal intensity in each frame [8]. This was done on the ten-times downsampled images, where the maximum value of ten voxels are taken as the value at each voxel position in each coordinate direction. Then the binary barcode $\hat{\mathbf{B}}_{r,c,j}$ from the original linear programming problem above can be scaled as $\alpha_{r,c} \cdot \hat{\mathbf{B}}_{r,c,j}$ to incorporate the frame-wise signal intensity into the barcode information. These scaled barcodes can be then be used as input for the 'BarDensr_underapprox' problem.

A.3 Amplicon estimation and morphological reconstruction

Having estimated the barcode library, we must next estimate the amplicon locations for each barcode. Finally, we must use those amplicon locations to reconstruct the neuronal morphology, by 'connecting the dots' between each estimated amplicon 'dot.'

A.3.1 Constructing an 'evidence tensor' to match barcodes to voxels

To begin, it is convenient to compute, for each voxel and each barcode, some estimate of our confidence that this barcode appeared in this voxel. We call an object of this kind an 'evidence tensor.' For the two simulations presented in this paper, we use two slightly different approaches to obtain such a tensor.

High-resolution, dense-expression simulation. In the high-resolution case, the signal is less mixed and the data is large; in this regime we found it sufficient to use a computationally cheap correlation method to compute the evidence tensor for each voxel and each barcode:

$$\text{cellratio}_{m,j} = \frac{\sum_{r,c:\ \mathbf{B}_{j,r,c}=1} \mathbf{X}_{r,c,m}}{\text{cellratio\_signal\_control} + \sqrt{\sum_{r,c} \mathbf{X}^2_{r,c,m}}}.$$

These ratios give a rough indication for whether a cell with barcode j might be present at voxel m. Where they are higher it suggests such a cell may be present. An example of the evidence tensor on a 2D plane is shown on Fig 7, where a good agreement between the correct neuronal shape and the structure of the evidence tensor is seen.

Note that although this metric is relatively fast to compute, to directly apply it to the entire array of high-resolution data (size of (146, 146, 197) voxels, which gives $M = 4,199,252$ voxels) for all neurons found (which gives $J = 975$ if all the barcodes are found) was not feasible because of memory constraints. Therefore, we used a simple tiling method and computed the evidence tensor for every discovered barcode within multiple small tiles with size of (30,30,30) voxels, with 10 voxels overlapping at each coordinate. We can further scale up the computation to even larger FOVs by additionally including the knowledge that there is only a small portion of the discovered barcodes present within each small tile.

Low-resolution, sparse-expression simulation. The simple correlation method above is insufficient analyzing low-resolution data; in this dataset the typical voxel has contributions from many barcodes, and the correlation method does not appear to be effective in this situation. Instead, we use 'amplicon density' as the evidence tensor, as described in [8]. Briefly, using BarDensr, the original imagestack $\mathbf{X}$ is demixed into two components - the amplicon density $\mathbf{F}$ and the accumulated barcode signal $\mathbf{G}$ (which is the scaled version of $\mathbf{B}$ mentioned in A.2.2):

$$\mathbf{X}_{r,c,m} \approx \sum_j \mathbf{G}_{r,c,j} \mathbf{F}_{m,j} + \text{background}.$$

Similar to $\text{cellratio}_{m,j}$, the amplicon density $\mathbf{F}_{m,j}$ at voxel m and barcode j indicates whether this barcode is present at this location.

A.3.2 Using the evidence tensor to reconstruct morphology

The evidence tensor constructed above is at best a noisy indicator of the neuron's morphology. On the one hand, there may be voxels inside the cell without any transcripts. On the other hand, there may be voxels influenced by several cells (due to PSF blurring, voxelization artifacts, errors in barcode or amplicon estimation, etc.). To use the evidence tensor to best advantage in estimating the final morphology, we consider two approaches.

First, we considered alphashape [11], which is a widely used approach to estimate solids from pointclouds. The method takes one parameter, α, used to balance over- and under-coverage of the resulting estimate. Visual inspection was used to select this parameter to obtain the best possible result. For high-resolution simulations we used $\alpha = 0.1$, and for low-resolution simulations we used $\alpha = 0.05$.

The second approach involves Convolutional Neural Networks (CNNs). We first estimated the evidence tensors for all the neurons in the volume and used them as the input to the CNN. The original data, after data augmentation (see below Appendix A.3.4), was split into the training and testing set. We then used the training set to train a neural network to reconstruct the true morphology of a single neuron from the evidence tensor for the corresponding barcode. We used a four-layered CNN with residual blocks structure [12]. The network was trained on 'total variation distance' as the target function:

$$\text{TV} = \frac{1}{2}\Big(\sum_m \left| \frac{y_m}{\sum_m y_m} - \frac{x_m}{\sum_m x_m} \right| \Big).$$

Here, x and y are either integer or float vectors. In our case, $y_m \in \{0, 1\}$ is the ground-truth label at voxel m and $x_m \in [0, 1]$ is the predicted label for m. TV is a distance between two probability measures. We also investigated two other losses: Wasserstein distance (which proved computationally expensive) and binary cross-entropy (which was less effective because the loss was dominated by large regions where no amplicons were present). In practice, TV proved efficient and effective. To train the network while ensuring the relevant rotational symmetries, we used data augmentation. More details can be found in Appendix A.3.4.

A.3.3 Evaluating performance

We use two different criteria to measure the performance of our methods. The first is the discovery rate, which quantifies the proportion of the neuron barcodes that we can identify. The second criterion was total variation distance, described above, a quantitative way to represent the accuracy of morphological reconstruction.

A.3.4 Tiling and Data Augmentation for Convolutional Neural Net training

To train neural networks to reconstruct morphology from evidence tensors in the high-resolution setting, we used the procedure discussed above to generate images of size $(60, 60, 60)$. We then augmented the data by taking six transformations of each tile: flipping over each axis, and swapping each pair of axes. All of these transformed versions of the data were fed as training data to the network at the same time, which helped ensure the network learned a function which was invariant to these kinds of transformations.

For network training in the low-resolution setting, we generated images of size $(40, 27)$, and augmented this data with three transformations: flipping over each axis and swapping the two axes.

A.3.5 Euclidean Distance Transform (EDT)

For low-resolution simulation, we used Euclidean Distance Transforms (EDT) to calculate the largest sphere that could be contained inside a neuronal segment, which is then used to determine whether a given segment was dendrite or axon; if a segment can contain a sphere with radius of 5 voxels (25 microns), we categorized it to be dendrite.

The Euclidean Distance Transform is defined as follows. One begins with a 2D or 3D image f, and let S be the set of voxels in the image f whose values are 1. Following [13], the distance map of the input image f is defined as

$$\mathrm{EDT}_p[f] = \min_{p \notin S} \|p - q\|^2,$$

where $\|p-q\|^2$ is Euclidean distance between voxel locations p and q. The computational cost for computing this transform scales linearly with the number of voxels in the image [13].

References

[1] S. Dorkenwald, N. L. Turner, T. Macrina, K. Lee, R. Lu, J. Wu, A. L. Bodor, A. A. Bleckert, D. Brittain, N. Kemnitz *et al.*, "Binary and analog variation of synapses between cortical pyramidal neurons," *BioRxiv*, 2019.

[2] C. M. Schneider-Mizell, A. L. Bodor, F. Collman, D. Brittain, A. A. Bleckert, S. Dorkenwald, N. L. Turner, T. Macrina, K. Lee, R. Lu *et al.*, "Chandelier cell anatomy and function reveal a variably distributed but common signal," *bioRxiv*, 2020.

[3] P. Zhou, J. Reimer, D. Zhou, A. Pasarkar, I. A. Kinsella, E. Froudarakis, D. Yatsenko, P. Fahey, A. Bodor, J. Buchanan *et al.*, "Ease: Em-assisted source extraction from calcium imaging data," *bioRxiv*, 2020.

[4] J. M. Kebschull and A. M. Zador, "Cellular barcoding: lineage tracing, screening and beyond," *Nature methods*, vol. 15, no. 11, pp. 871–879, 2018.

[5] J. M. Kebschull, P. G. da Silva, A. P. Reid, I. D. Peikon, D. F. Albeanu, and A. M. Zador, "High-throughput mapping of single-neuron projections by sequencing of barcoded rna," *Neuron*, vol. 91, no. 5, pp. 975–987, 2016.

[6] Y.-C. Sun, X. Chen, S. Fischer, S. Lu, H. Zhan, J. Gillis, and A. M. Zador, "Integrating barcoded neuroanatomy with spatial transcriptional profiling enables identification of gene correlates of projections," Nature Publishing Group, Tech. Rep., 2021.

[7] X. Chen, Y.-C. Sun, H. Zhan, J. M. Kebschull, S. Fischer, K. Matho, Z. J. Huang, J. Gillis, and A. M. Zador, "High-throughput mapping of long-range neuronal projection using in situ sequencing," *Cell*, vol. 179, no. 3, pp. 772–786, 2019.

[8] S. Chen, J. Loper, X. Chen, A. Vaughan, A. M. Zador, and L. Paninski, "Barcode demixing through non-negative spatial regression (bardensr)," *PLoS computational biology*, vol. 17, no. 3, p. e1008256, 2021.

[9] M. Tepper and G. Sapiro, "Nonnegative matrix underapproximation for robust multiple model fitting," in *2017 IEEE Conference on Computer Vision and Pattern Recognition (CVPR)*, 2017, pp. 655–663.

[10] H. Inan, C. Schmuckermair, T. Tasci, B. Ahanonu, O. Hernandez, J. Lecoq, F. Dinç, M. J. Wagner, M. Erdogdu, and M. J. Schnitzer, "Fast and statistically robust cell extraction from large-scale neural calcium imaging datasets," *bioRxiv*, 2021.

[11] H. Edelsbrunner, D. Kirkpatrick, and R. Seidel, "On the shape of a set of points in the plane," *IEEE Transactions on information theory*, vol. 29, no. 4, pp. 551–559, 1983.

[12] K. He, X. Zhang, S. Ren, and J. Sun, "Deep residual learning for image recognition," in *Proceedings of the IEEE conference on computer vision and pattern recognition*, 2016, pp. 770–778.

[13] P. F. Felzenszwalb and D. P. Huttenlocher, "Distance transforms of sampled functions," *Theory of computing*, vol. 8, no. 1, pp. 415–428, 2012.
