# Supplementary figures and images for: Blind demixing methods for recovering dense neuronal morphology from barcode imaging data
Source: PLoS Comput Biol. 2022 Apr 8;18(4):e1009991. doi: 10.1371/journal.pcbi.1009991 (PMC9020678; doi:10.1371/journal.pcbi.1009991)

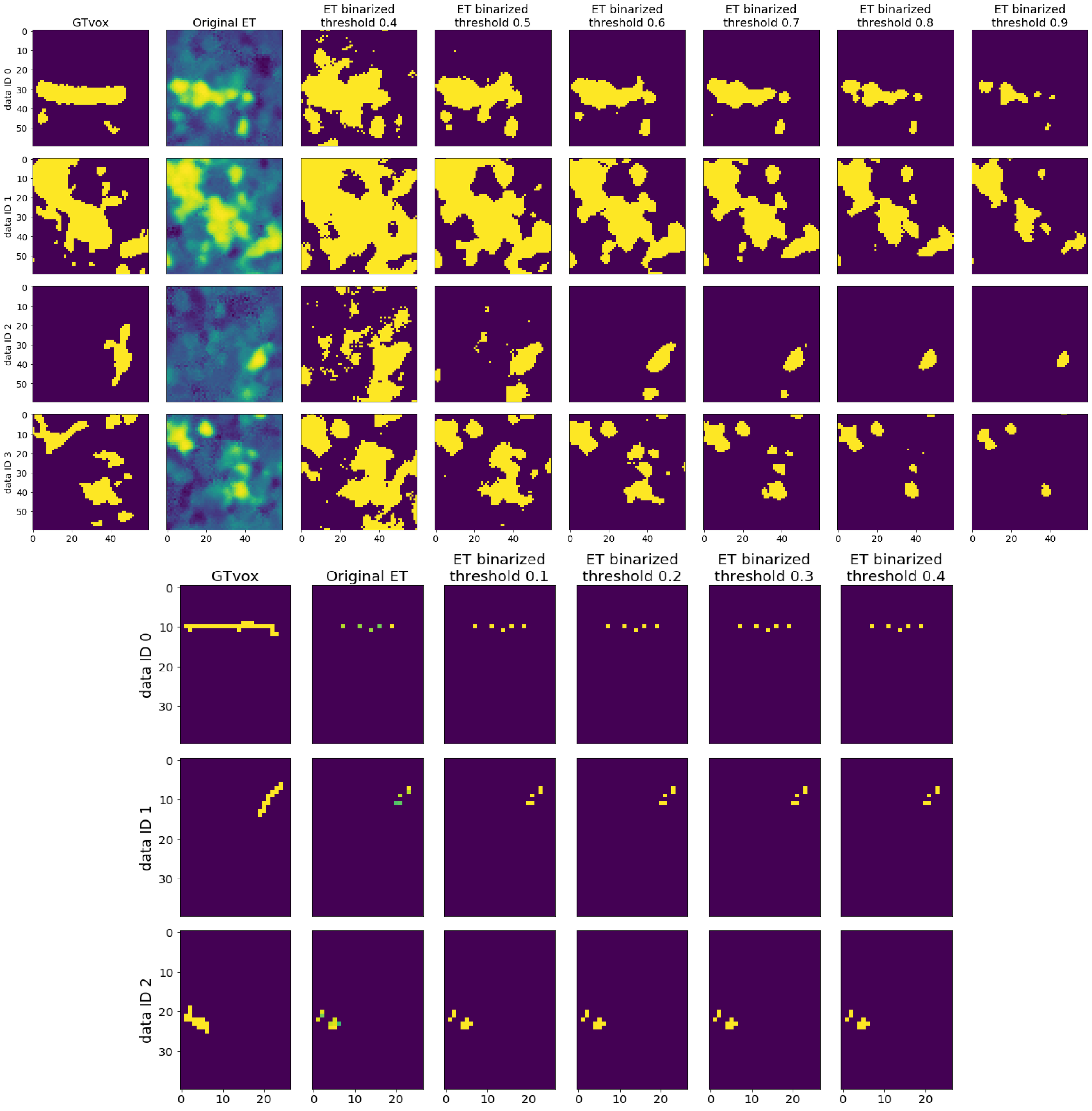

Supplement: S1 Fig — In order to create input data for the morphology prediction in Figs 8–11, we needed to binarize the evidence tensors. Here we show the test cases (one row shows one example neuron) where we used various thresholds to binarize the evidence tensors (the third columns and the following) and compared the result with both original ground-truth voxels (first columns), as well as the original (continuous) evidence tensor (second columns). For the high resolution simulation, we used 0.7 as the threshold since the result was robust. This threshold was used to visualize Figs 8 and 9. For the low resolution simulation, we used BarDensr to estimate the evidence tensor and the results were much sparser than the high resolution case, and therefore the binarization was relatively robust to the choice of the threshold. Here we used 0.1 for the low resolution case. This threshold was used to visualize Figs 10 and 11. (TIFF) [file pcbi.1009991.s002.tiff]

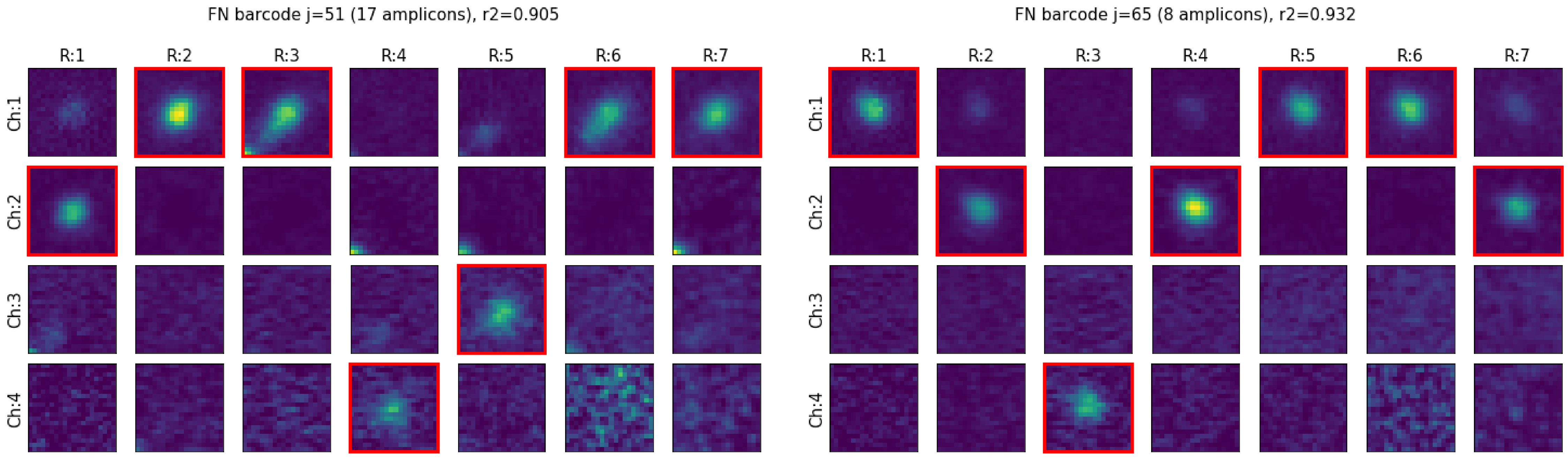

Supplement: S2 Fig — Here we show two examples of the amplicons that are from the barcodes that were not found in our amplicon detection process in Fig 6). For both panels, the red frames indicate where the correct barcode signals are expected. The left panel shows the missed barcode which had the most abundant amplicon. We see that the signal intensity varies significantly (e.g., round 2 has much higher signal compared to round 4 and 5). The right panel showed missed barcodes with the third most abundant amplicons. From round 2, 4 and 7 we see there is a relatively high phasing and/or color-mixing happening in the channel 1, which might have made the barcode discovery difficult. (TIFF) [file pcbi.1009991.s003.tiff]
