# Supplementary material for: Blind demixing methods for recovering dense neuronal morphology from barcode imaging data
Source: PLoS Comput Biol. 2022 Apr 8;18(4):e1009991. doi: 10.1371/journal.pcbi.1009991 (PMC9020678; doi:10.1371/journal.pcbi.1009991)
Supplement: Attachment — Submitted filename: coverletter_revision.pdf [file pcbi.1009991.s004.pdf]

February 7, 2022

Dear Editor-in-Chief,

We thank the reviewers for their constructive feedback on our manuscript. We have revised the manuscript significantly to clarify the points that were raised by the reviewers.

In addition to these significant revisions, we added a new figure (Figure 6, please note the change in the indices of the following figures), showing our method's performance on a real experimental dataset. We used this data to quantify the performance of the iterative barcode discovery method proposed in this paper. This figure may also help readers compare our simulation data to actual experimental data.

There were also several other notable points, which we address below. All changes in our new manuscript (attached) are highlighted with purple.

Again, thank you for your consideration and we look forward to hearing from you.

Sincerely,
Shuonan Chen

Reviewer 1

"I find the statements on the spatial extent (projection pattern in the abstract, etc) a bit overreaching. The authors actually use a careful language (e.g., "suggest the possibility"). However, the paper does not at all concern itself with scaling in space. Why should we think that this algorithm would work when the volume is millions of times larger, which is what's needed for those claims? If the approach is to divide-and-conquer, how could we merge the subvolumes? (e.g., gluing the interface may not be straightforward.) I find the lack of at least a careful discussion of questions like these an important omission."

Thank you for this suggestion. We have added some comments in the Conclusion section regarding this challenge. More investigation is certainly needed; however, it may be premature to make too many claims about how this will work out before the proposed experiments are performed.

"The results feel somewhat anecdotal and appear to be based on a single simulation. Could the authors characterize the uncertainty by reporting e.g., mean & st dev values over a few simulation runs? Easiest could be to use the same EM volume while simulating the barcode assignments from scratch. Also, using more than one EM volume might not be hard because multiple such volumes were published in the past few years."

Thank you for this suggestion. We now have added two new experiments, one on the same volume but different barcodes, and another on a different volume, and we have updated Figure 5 with the mean and the standard deviation.

"I don't think the paper's TV loss implementation is a common choice for similar computer vision tasks. Please better explain this choice and/or provide references."

We are not aware of any typical computer vision tasks which closely match ours, i.e. morphological reconstruction from a noisy indicator for sparse signals. There is certainly an open question as to what formal losses may be best for purposes of achieving satisfactory reconstructions. We investigated several other losses in our journey to get to this point, including binary cross-entropy losses (ineffective), Wasserstein losses (computationally too intensive), and Chamfer distances (non-differentiable). For now, total variation

appears to be the best option. We have now added some discussion on this point.

"- "In all cases we chose thresholds to ensure at most 3 false positives." I find the y-axes being absolute counts somewhat inappropriate for the claims of this paper. Could you please present them as false positive rates? (e.g., count/volume, count/neuron) Also, I couldn't understand if this rate is supposed to be representative or minimal - please explain how you chose 3. Lastly, the definition of the threshold is not clear at this point in the main text."

Thank you for the suggestion. We found it helpful to look at the absolute number of false positive barcodes identified in a given slice of tissue with a given number of infected neurons at a given amplicon density. We hope this number will give experimentalists the fullest sense of what to expect. Your suggestion lead to two useful improvements to the paper. First, we have now added plots showing precision (i.e. true positives divided by total predicted barcodes), which gets at the notion of the false positive rate. Second, we have added some remarks about how the threshold was picked to the first results section where barcode estimation is considered.

"- The main text seems to target biologists and defers all technical details to the Methods section. This style is somewhat justified in the presence of Ref. [16]. However, as a stand-alone paper, it does not adequately communicate the technical context for a comp. bio. paper: (a) The main idea behind the algorithm of Ref. [16] could be described in more detail in the main text. (b) The differences (add-ons) of the present paper with the algorithm of Ref. [16] should be stated in the main text. (c) How does this paper relate to/compare with the group's previous approach on a similar subject; Sumbul, . . . , Paninski, "Automated scalable segmentation of neurons from multispectral images", 2016?"

We have added the description of Ref. [16] in Introduction section, and elaborated on the idea of BarDensr as well as its differences from this work. Lastly, the work by Sumbul (Ref [39] in the new manuscript) is added and discussed where we talked about the Brainbow work on Related work section.

"- Similar to the previous comment, a central claim is the "novel blind-demixing algorithm", but this is barely explained in the main text. Again, while I somewhat understand the authors' intention, I think the main text could do more in this direction. (e.g., what's novel about it? what's better about it? what makes it suitable for the problem?)"

We have included some sentences in Introduction section to address this point. We have elaborated why this blind-demixing algorithm is important and enables the other analysis. Regarding novelty, the algorithm is novel mainly insofar as it tackles a problem hereto uninvestigated. Naive blind-demixing algorithms which do not take advantage of the spatial structure in this problem are completely ineffective. We have added some remarks on this point to the Barcode estimation section (in Method summary).

"- Fig. 8 & Fig. 10: GT and CNN recons. differ quite a bit. Why is the presented accuracy good enough? Would they look much more similar if a slab (a few slices) were projected? As is, I do not get the sense that whole neuronal morphologies can be recovered faithfully. Since this is a central claim, presentation and interpretation of the results should be much more careful."

CNN reconstruction is indeed not perfect in Figure 9 and 11 (previously Figure 8 and Figure 11). Figure 9 (previously Figure 8, high resolution) is slightly better, but for Figure 11 (previously Figure 10, low resolution) the reconstruction indeed could have been improved. Quantitatively (bottom panel of Figure 9 and Figure 11), we see that CNN has better performance than the simple linear approach such as alphashape. Qualitatively, the results are hard to judge. To help readers make their own judgements about whether we have achieved "faithful reconstruction," as suggested by the reviewer, we have changed the visualization of Figure 9 from one z-plane to the sum-projection across all z-planes. We think this gives better information in terms of reconstruction accuracy, and indeed, CNN has slightly better performance. Ultimately the question of whether our morphological reconstructions are "good enough" can only be evaluated in the context of a particular task, but in this work we sought only to explore the feasibility in a general sense (to encourage experimentalists); in future work we hope to investigate these issues more closely.

"- Including a few representative BARseq slices or referring to specific figures in other papers could make it easier to visually assess the level of realism of the simulations."

Good idea; we have added Figure 6 showing our blind-demixing performance on the real data, and compared the real data with the simulated data on the top panel. Note that this real data does not exactly correspond to STIBE data; the targets are gene transcripts (and that is why we see different colors within a single cell). But at least the readers can get a sense of how similar our simulation is compared to the real data.

"- Fig. 3, right, appears saturated."

We have adjusted the intensity for this right panel, thank you.

"- line 238: As shown in the *bottomn* ..."

Fixed, thank you.

"- line 378: $\tilde{B}'-->\tilde{B}$ (Please check the prime.)"

Fixed, thank you.

Reviewer 2

[Major] "If I understand correctly (the description is a bit short), the CNNs for shape recovery have been trained on the same data that was used for the experiments. That means that it could have learned the shapes of the neurons that it later aims to recover. If that is true, this experiment should be repeated with training on data that is not used for the demixing experiment, either from a different part of the volume or from neurons that are then removed from the dataset."

Thanks for clarifying this important point. In fact, we did training and testing on different datasets. We have included this discussion to clarify the point in the Appendix A.3.2.

"11-12 Either or makes no sense here, low resolution limits the number more rather than less"

We rephrased it, to express that in the case of traditional molecular techniques combined with light microscopy, often times we have limited resolution, and even when this can be ignored (for instance, we may just want to map the rough pattern of the shapes), it is still challenging to map many cells at once.

"47 I don't understand why high density in the high-resolution regime makes reconstruction harder. Can you explain? In the low-resolution it is obvious, because all voxels have arbitrarily mixed signals, so the iterative unmixing cannot start anywhere, and fine processes would remain mixed after many iterations."

We agreed with this point and have rephrased it by emphasizing the 'limited resolution'. The problem is this: even our "high resolution" case (which approaches the limits of experimental feasibility) does not have high enough resolution to make all the demixing problems trivial.

"Figure 4 (and others) the GT neuron morphologies are rendered as if each voxel were occupied by only one neuron. You make the point that this is not the case which makes sense because neurons have thin processes and the reconstruction comes from high resolution EM. Can you render them by mixing the colors according to voxel coverage? You could do that by voxelizing them at e.g. 4x the target resolution, then downsample with are aaveraging. That would make the isseu more obvious."

We have updated all the figures that are relevant, by mixing the colors of neurons whenever there are overlaps in the voxels (we did not need to do the downsampling since we have the original voxel location for each neuron). This makes the visualization much clearer and indeed makes the issue more obvious. Thank you for the suggestion. The affected figures include Figure 3, 4, 8 (previously 7) and 10 (previously 9), for both high and low resolution simulation.

"Figure 4 The amplicon locations in the bottom panels are consistently between voxels instead of in voxel

centers. The Gaussian amplicon spots look as if they are centered at voxels, not between voxels. I do not understand what that means and also do not understand how that leads to color mixing of up to 6 neurons. Can you please make this more clear?"

Fixed - it was a visualization error and should both be centered at voxels. Thank you for pointing out.

"Figure 5 Please use non-stacked mulit-histogram plots for the first two plots. This will make the hidden results in the second plot visible and the iterations axis is not continuous anyways."

Thank you for the suggestions. We have modified Figure 5 to include more simulation replicates. Instead of multi-histogram, we have kept the line plots and added visualizations for the standard deviation. We tried multi-histograms with error bars but found the plot too busy to be clear. Although the iteration axis is not continuous, the line plots are essential for visualizing the improvement over time.

"Figure 7 Why are the recovered amplicons so big? Wouldn't it be better to detect their center points? Or are they actually that big and so they can fit only where the neuron has a caliber of about 1um? Cna you discuss this briefly?"

Thank you for pointing this out. The apparent size of the recovered amplicons in Figure 8 (previously Figure 7) is largely a function of the point-spread-function in the simulated optics. We have clarified this point.

"Figure 8 May be 3D renderings of the shapes could be more informative but not sure. Slices are often misleading about the true nature of differences because 3D context is missing. But again, you may have tried this and it was worse? Also, again, why did you choose to threshold the fuzzy amplicon images instead of localizing them? Does it work better?"

We agree that the slices are slightly misleading in this case. We have changed from one z-plane to the sum-projection over all z-planes so that we can better visualize our prediction performance. (Also see the video across z-planes.) In terms of the thresholded fuzzy amplicons, we did carefully choose the threshold in order to match the shape of the binarized evidence tensor to the original amplicon locations, and we have added examples in Figure S1 (supplementary), since this was also asked later in the comments. We could not find a straightforward way to localize the exact amplicon locations based on the learned evidence tensor; one may sometimes have three or more amplicons clustered together, making it nontrivial to discern exact locations.

"234–241 The entire section 4.2.1 is written as if the paper before didn't really exist and is confusing, can you please revisit this? May be giving the naive approach and the iterative approach a name, and not call the same iterative method "an iterative approach" but "the iterative approach"?"

We do think calling 'the iterative approach' is much better and less confusing than 'an iterative approach' since this is essentially the same as the one used in the high-resolution case. This was changed, thank you for the suggestion.

"264 "like improved methods" is probably a typo"

Thank you for the suggestion; it was not a typo but it was a bit confusing. We have edited it.

"Figure 9 Like in Figure 4, it would be much clearer to see how neurons partially occupy individual voxels. Probably more important here than in the high-resolution experiment. Can you voxelize at a higher resolution and then resize with averaging?"

Fixed as in Figure 4, and we believe this is a much better visualization, thank you.

"Figure 9 The red boxes in the simulated image are practically invisible, may be put a thicker frame around them?"

Fixed, thank you.

“Figure 10 How did you decide that the evidence tensor should be thresholded at 0.1 (low-resolution) and 0.7 (high-resolution), respectively?”

Thank you, we have added a supplementary figure to clarify this process. Specifically, these are based on the visual inspection of binarized evidence tensor (at each threshold) and the ground-truth amplicon locations. We have added more detail in the caption of Figure S1.

“Algorithm 1 4 The indices r,c are not used inthe foreach loop and can be removed.”

“7, 8 none of the indices are used and so they can be removed”

Fixed; we have updated Algorithm 1 by removing the unnecessary indices and adding for loops when necessary to make it clearer.

“Algorithm 2 index j is not used and can be removed”

Similar to Algorithm 1, we have removed the unnecessary indices and added new letter (β that indicates codebook for a particular barcode) for clarity. We believe both these two algorithms are clearer now. Thank you for the suggestions.

“A.1.1 and A.1.2 As in Figures 4 and Figure 9 I suggest to voxelize at much higher resolution, then down-sample with area averaging to mix colors (or mix colors maively)?”

We have now updated figures to show the mixed colors.

“Algorithm 3 each iteration is affected by noise and I would expect that noise to accumulate with more iterations. Is that a valid concern? May be not important with low iteration counts as in these experiments.”

Thank you for raising this point. It is possible that the noise accumulates over the iterations. This could be one of the reasons that the iterative method did not have perfect performance on the real experimental data (as shown in the newly added Figure 6). At this point we do not have a satisfactory answer.
